# Strength Training Intensity and Volume Affect Performance of Young Kayakers/Canoeists

**DOI:** 10.3389/fphys.2021.686744

**Published:** 2021-06-24

**Authors:** Martijn Gäbler, Hermine S. Berberyan, Olaf Prieske, Marije T. Elferink-Gemser, Tibor Hortobágyi, Torsten Warnke, Urs Granacher

**Affiliations:** ^1^Department of Human Movement Sciences, University Medical Center Groningen, University of Groningen, Groningen, Netherlands; ^2^Division of Training and Movement Sciences, Research Focus Cognitive Sciences, University of Potsdam, Potsdam, Germany; ^3^Bernoulli Institute for Mathematics, Computer Science and Artificial Intelligence, University of Groningen, Groningen, Netherlands; ^4^Division of Exercise and Movement, University of Applied Sciences for Sports and Management Potsdam, Potsdam, Germany; ^5^Research Group Canoeing, Institute for Applied Training Science, Leipzig, Germany

**Keywords:** youth sports, water sports, exercise test, athletic performance, anthropometry

## Abstract

**Purpose:**

The aim of this study was to compare the effects of moderate intensity, low volume (MILV) vs. low intensity, high volume (LIHV) strength training on sport-specific performance, measures of muscular fitness, and skeletal muscle mass in young kayakers and canoeists.

**Methods:**

Semi-elite young kayakers and canoeists (*N* = 40, 13 ± 0.8 years, 11 girls) performed either MILV (70–80% 1-RM, 6–12 repetitions per set) or LIHV (30–40% 1-RM, 60–120 repetitions per set) strength training for one season. Linear mixed-effects models were used to compare effects of training condition on changes over time in 250 and 2,000 m time trials, handgrip strength, underhand shot throw, average bench pull power over 2 min, and skeletal muscle mass. Both between- and within-subject designs were used for analysis. An alpha of 0.05 was used to determine statistical significance.

**Results:**

Between- and within-subject analyses showed that monthly changes were greater in LIHV vs. MILV for the 2,000 m time trial (between: 9.16 s, SE = 2.70, *p* < 0.01; within: 2,000 m: 13.90 s, SE = 5.02, *p* = 0.01) and bench pull average power (between: 0.021 W⋅kg^–1^, SE = 0.008, *p* = 0.02; within: 0.010 W⋅kg^–1^, SE = 0.009, *p* > 0.05). Training conditions did not affect other outcomes.

**Conclusion:**

Young sprint kayakers and canoeists benefit from LIHV more than MILV strength training in terms of 2,000 m performance and muscular endurance (i.e., 2 min bench pull power).

## Introduction

Increasing athletic and sport-specific performance requires an optimization of the training stimulus. Cold weather is an impediment for young kayakers and canoeists, as specific (on-water) training is halted for up to 5 consecutive months per year, depending on geographical location. One way to compensate for the missed on-water training is to emphasize the improvement of physical fitness through event-specific exercises. There is evidence that measures of muscular fitness [i.e., muscle strength (0.5 ≤ *r* ≤ 0.9), muscle power (0.6 ≤ *r* ≤ 0.7), muscular endurance (0.5 ≤ *r* ≤ 0.9)] maximum oxygen uptake (0.6 ≤ *r* ≤ 0.9), and skeletal muscle mass (0.4 ≤ *r* ≤ 0.9) are associated with kayak/canoe performance in kayakers competing at regional to international level (Fry and Morton, 1991; Van Someren and Howatson, 2008; Forbes et al., 2009; Hamano et al., 2015). As a common element across regression models (0.66 ≤ *R*^2^ ≤ 0.87), work generated during ergometer or bench pull exercises for 30–120 s on land might be an accurate predictor of on-water performance (200–2,000 m) in kayakers and canoeists from regional to international competitive level (Van Someren and Howatson, 2008; Gäbler et al., 2021). The combination of strength and endurance training (i.e., concurrent training) thus seems highly effective for kayak/canoe athletes, as it can simultaneously improve muscular and cardiorespiratory fitness (Wilson et al., 2012) and it can improve athletic performance in endurance athletes more than endurance training alone (Gäbler et al., 2018).

Several factors may influence the adaptations to concurrent training, including the intensity of strength training (Docherty and Sporer, 2000). Athletes improve muscle strength more when strength training is performed at intensities above 70% of the one repetition maximum (1-RM) (0.6 ≤ $\bar{\text{ES}}$ 1.1) than at lower intensities (0.1 ≤ $\bar{\text{ES}}$ 0.2) (Peterson et al., 2004). Larger effect sizes were observed at training intensities above 85% 1-RM ($\bar{\text{ES}}$ 2.5) with six to eight repetitions ($\bar{\text{ES}}$ 2.4) in young athletes (Lesinski et al., 2016). Moderate to high intensity (>70% 1-RM), low volume (MILV) strength training could be well suited for young kayakers and canoeists, because it increases muscle strength and hypertrophy, which are both associated with canoe and kayak performance (Fry and Morton, 1991; Van Someren and Howatson, 2008; Forbes et al., 2009; Hamano et al., 2015). However, anecdotal evidence indicates that German top-level kayak/canoe coaches prefer low intensity, high volume (LIHV) strength training. The metabolic and neuromuscular demands during competition resemble those during LIHV strength training. More specifically, in canoe sprint, the cyclical pulling motion is repeated at a stroke rate of 54–156 strokes per minute (Zahálka et al., 2011; Nilsson and Rosdahl, 2016) over the course of roughly 30 s to 30 min, depending on the event. Accordingly, the principle of training specificity (Häkkinen et al., 1989) suggests performing LIHV strength training, particularly to improve performance in longer distance trials.

The conflicting results in the literature make it difficult for practitioners to decide whether MILV or LIHV strength training is the most beneficial to develop young kayakers’ and canoeists’ performance. Therefore, the aim of this study was to compare the effects of MILV vs. LIHV strength training on sport-specific performance, measures of muscular fitness, and skeletal muscle mass in young kayakers and canoeists. In line with the principle of training specificity, we evaluate the hypothesis that training adaptations are specific to the intensity and volume of the training. Thus, we expect greater increases in short distance (250 m) kayaking/canoeing performance, measures of strength, power and skeletal muscle mass after MILV compared with LIHV training and greater increases in long distance (2,000 m) kayak/canoe performance and measures of muscular endurance after LIHV training compared with MILV.

## Materials and Methods

### Study Design

**FIGURE 1:**
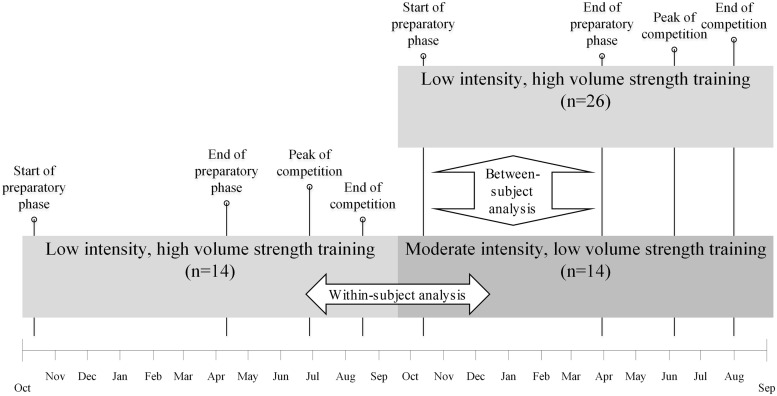
Graphic of study design. The boxes indicate when training conditions were performed on the time scale below. Vertical lines indicate the timing of measurement occasions and the corresponding seasonal phases.

The non-randomized trial took place over two consecutive seasons between 2016 and 2018 (Figure 1). Participants performed either MILV or LIHV strength training for an entire season in their predetermined training group. Fourteen participants completed both conditions across two seasons, whereas 26 participants performed only the LIHV condition in either season 2016/2017 or 2017/2018. This design allowed for both between- and within-subject comparisons. The primary outcomes were time trials (250 and 2,000 m), bench pull average power, underhand shot throw, handgrip strength, and skeletal muscle mass. Tests were realized during each season at the start of the preparatory phase (October) and at the end of the preparatory phase (March/April). In addition, hand grip strength and skeletal muscle mass were measured at the peak of the competitive phase (June/July) and at the end of the competitive phase (August). Assessors were blinded to participants’ group assignments.

### Participants

Semi-elite, young sprint kayakers and canoeists (*N* = 40, Table 1) volunteered to participate in this study. The athletes trained on average 12 h/week at the Olympic testing and training center in Potsdam, Germany. All participants competed at a regional or state level and were classified as semi-elite (Swann et al., 2015). Athletes and their legal guardians provided written informed consent prior to the start of the study. The study was performed in line with the declaration of Helsinki and approved by the University of Potsdam ethics committee (submission No. 26/2014).

**TABLE 1:** Participant characteristics at baseline.

	MILV	LIHV	Total
N boys	7	22	29
N girls	7	4	11
N kayak	3	13	16
N canoe	11	13	24
Chronological age (y)	13.1 ± 0.53	12.9 ± 0.92	13.0 ± 0.80
Maturity offset boys (y)^1^	−0.26 ± 0.72	−0.44 ± 1.15	−0.40 ± 1.06
Maturity offset girls (y)^1^	1.64 ± 0.52	0.97 ± 1.3	1.39 ± 0.88
Body height (cm)	166 ± 7.95	165 ± 10.1	165 ± 9.37
Body mass (kg)	52.1 ± 5.54	50.8 ± 10.7	51.2 ± 9.27
Relative fat mass (%)	12.9 ± 4.62	10.8 ± 4.91	11.5 ± 4.86
Skeletal muscle mass (kg)	25.0 ± 2.83	24.9 ± 5.5	24.9 ± 4.73
Bench pull average power (W⋅kg^–1^)	1.10 ± 0.17	1.12 ± 0.21	1.11 ± 0.20
Hand grip strength (N)	283 ± 31.0	281 ± 74.8	281 ± 63.1
Shot throw (m)	9.22 ± 1.22	8.09 ± 1.41	8.50 ± 1.44
Time trial 250 m (s) kayak	71.8 ± 2.76	68.9 ± 4.63	69.4 ± 4.41
Time trial 250 m (s) canoe	77.7 ± 4.95	81.0 ± 15.4	78.9 ± 9.81
Time trial 2,000 m (s) kayak	693 ± 9.90	690 ± 53.4	690 ± 49.6
Time trial 2,000 m (s) canoe	763 ± 48.3	838 ± 152	796 ± 110

*^1^Maturity offset was estimated using the equation proposed by Mirwald et al. (2002).*

### Primary Outcomes

Time trials of 250 and 2,000 m on an open, flatwater course were included as measures of sport-specific performance twice each season. The 250 m trial was performed in a 1 vs. 1 race on a straight course with two lanes. The 2,000 m trial was performed by going back and forth on a straight 1,000 m course. Thirty-second intervals separated the start of individual athletes. From our own data, using two-way mixed effects models for single rater measurements (Koo and Li, 2016), we estimated the test-retest reliability as excellent for both outcomes [intra-class correlation (ICC) ≥ 0.93).

We assessed 2-min bench pull average power as an outcome of muscular endurance twice each season. Trainers and athletes selected the resistance together, aiming to maximize individual total work. A wire connected the barbell to a displacement transducer to register the total distance covered. We calculated as follows the outcome average  power[$\bar{W}$] = $\frac{\mathrm{distance}\mathrm{covered}\left[m\right]\cdot \mathrm{barbell}\mathrm{mass}\left[\mathrm{kg}\right]\cdot g\left[{\mathrm{ms}}^{2}\right]}{\mathrm{time}\left[s\right]}$ that was previously identified as a predictor of 500 and 2,000 m canoe and kayak race time (Gäbler et al., 2021). We estimated the test-retest reliability (Koo and Li, 2016) as excellent for this outcome (ICC = 0.96).

The underhand shot throw was included as a measure of muscle power twice per season. We measured the distance athletes could throw a shot of 3 kg. The best of three trials was selected for further analysis. We estimated test-retest reliability (Koo and Li, 2016) as high (ICC = 0.85) from our own data.

We included maximum isometric handgrip strength of the dominant (writing) hand as an outcome of muscle strength four times per season using a handheld dynamometer (Jamar plus+ digital hand dynamometer, Patterson Medical, Cedarburg, WI, United States). The best of three trials was selected for further analysis. The test-retest reliability for the Jamar plus+ digital hand dynamometer was excellent (0.93 ≤ ICC ≤ 0.96) (Guerra et al., 2017).

Skeletal muscle mass was assessed four times per season using bioelectrical impedance analysis with an octopolar tactile-electrode impedance meter (InBody 720, Biospace, Seoul, South Korea) in the morning while fasted. Concurrent validity with dual energy X-ray absorptiometry and test-retest reliability were excellent (ICC ≥ 0.89) for all outcomes (McLester et al., 2018).

### Training

Training time (12 ± 2.9 h/week) was divided into strength training (25%), non-specific endurance training such as running and swimming (20%), event-specific endurance training (25%), and games and calisthenics (30%). Participants performed three strength training sessions per week in the morning on Monday, Wednesday, and Friday. Half of the strength training was devoted to the main exercises, i.e., bench pull and bench press. The remainder of the strength training was the same for all participants and was devoted to a range of varying exercises such as pull-ups, dips, squats, deadlifts, arm curls, Russian twists, and planking. Other training was kept constant for all participants as well.

In the MILV condition, participants performed the main exercises at moderate-to-high intensity (i.e., 70–80% 1-RM) and low volume (i.e., 6–12 repetitions per set over 3–4 sets). Every 5–10 weeks, 1-RM measures were taken to ensure progression of intensity. 1-RM was quantified as the greatest mass the athlete could lift with proper form within five attempts. In the LIHV condition, participants performed the main exercises at low intensity (i.e., 30–40% 1-RM) and high volume (i.e., 60–120 repetitions over two sets), integrated in a circuit with other exercises.

### Statistical Analyses

Due to the longitudinal design, we had missing values of the study that were completely at random (Little and Rubin, 2019). Participants with fewer than two data points for an outcome were removed from further analysis. For each analysis, we removed data points that were beyond two standard deviations of the mean (Berberyan et al., 2021). This “outlier” detection procedure was performed as outliers tend to contaminate the results and may lead to statistical errors (i.e., Type-II error), particularly in smaller datasets (Cousineau and Chartier, 2010). On average, 2.05% of the data was identified as an outlier and removed. The average percentage of missing values in the outcome variables was 8.94%.

We constructed linear mixed-effects models (LME) to determine whether training condition (i.e., MILV or LIHV strength training) affects the changes over time in the primary outcomes (i.e., 250 and 2,000 m time trials, bench pull average power, shot throw, handgrip strength, and skeletal muscle mass). For each outcome, we performed both a between-subject analysis and a within-subject analysis. In the between-subject analysis, training conditions were performed by two independent groups of participants. The models for this analysis included the within-subject factor time (i.e., month of measurement occasion) and the between-subject factor training condition (i.e., MILV and LIHV strength training). In the within-subject analysis, training conditions were performed by the same group of participants. Therefore, the models for this analysis included training condition as a within-subject factor. As in the between-subject analysis, the factor time was also included as a within-subject factor.

linear mixed-effects models models were fitted in R (R Core Team, 2020) by means of lme4 package (Bates et al., 2015). For evaluating *p*-values from LMEs we used lmerTest package (Kuznetsova et al., 2017) by applying Satterthwaite’s approximation method. In both between- and within-subject analyses, three fixed effects have been tested: the main effect of time, the main effect of training condition, and their interaction term. Additionally, boat type (i.e., kayak/canoe) was included as a fixed effect in the analyses of the time trials. Alpha for fixed effect estimates was set at 0.05. To obtain the random effects structure that fits best to the data, we applied stepwise forward fitting. This was done due to the fact that random effects can also influence the generalizability of the findings (Barr et al., 2013). Thus, we aimed to obtain the maximal random and fixed effects structure that is supported by the data. Model coefficients were calculated by deploying parametric bootstrapping (1,000 simulations) as implemented in the bootMer function of the lme4 package.

Combining estimates from the current models with earlier predictive models (Gäbler et al., 2021) allows for an estimation of predicted changes in race performance over time. An increase of 0.1 W⋅kg^–1^ in average bench pull power corresponds to a decrease in predicted race time of 4.37 and 15.05 s in 500 and 2,000 m race time. Accordingly, an increase of 1 kg in skeletal muscle mass corresponds to a decrease in predicted race time of 1.32 s (500 m) and 6.80 s (2,000 m).

## Results

### Between-Subject Analysis

**FIGURE 2:**
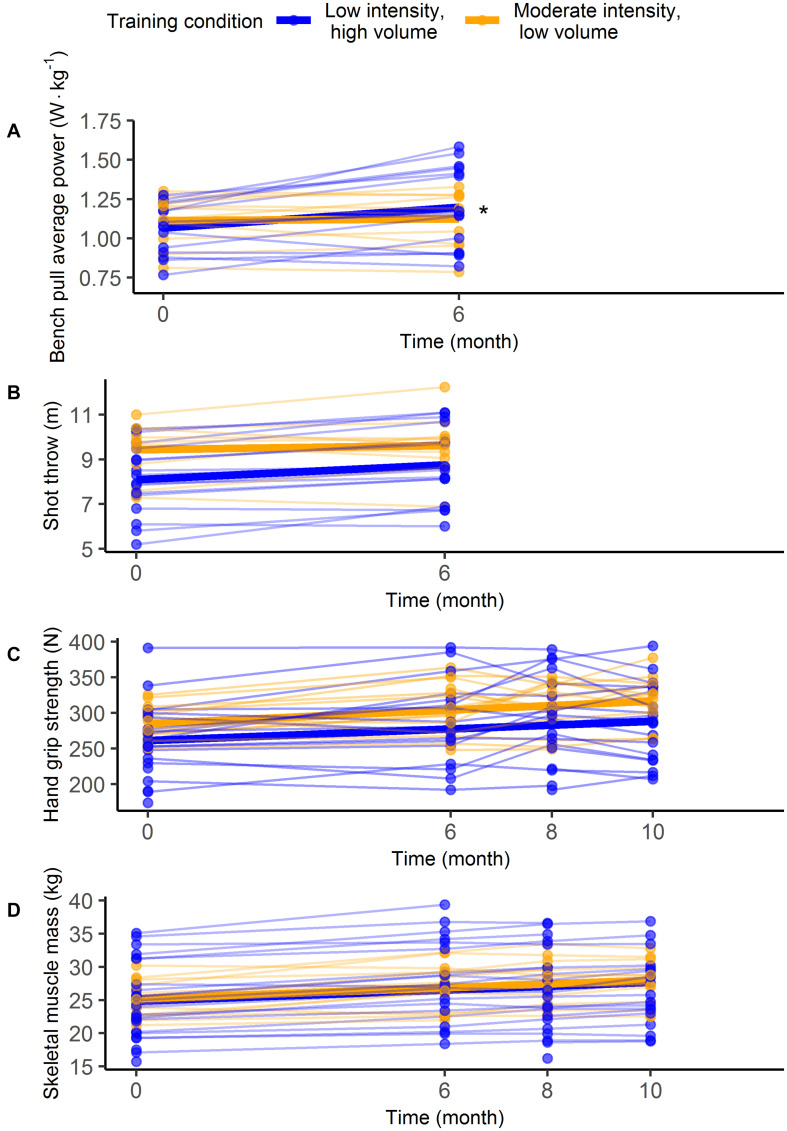
Individual data (thin lines) and model estimates (thick lines) of the between-subject analyses for the outcomes bench pull average power **(A)**, shot throw **(B)**, hand grip strength **(C)**, and skeletal muscle mass **(D)**. Asterisks (*) flag significant interactions.

**FIGURE 3:**
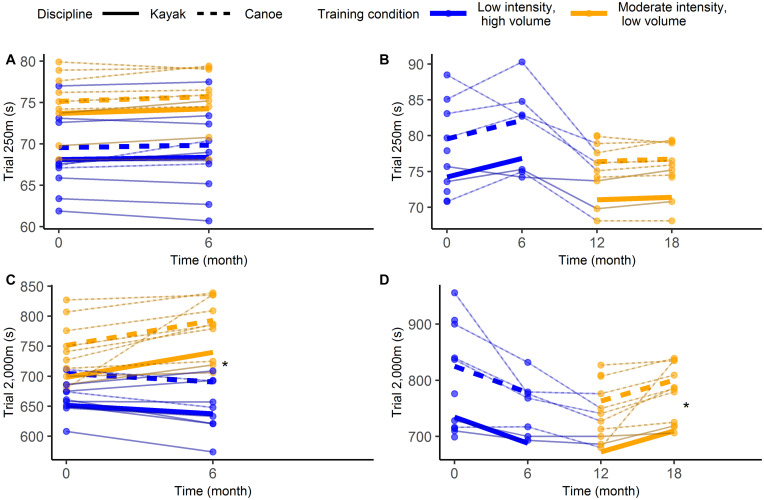
Individual data (thin lines) and model estimates (thick lines) for on-water performance for the between-subject analyses **(A,C)** and the within-subject analyses **(B,D)**. Asterisks (*) flag significant interactions.

Measures of muscular fitness and skeletal muscle mass increased significantly over time (*t* ≥ 4.2, *p* < 0.001), but no main effects of time were found for the time trials. Significant time by training condition interactions were present in the outcomes bench pull average power (*t* = 2.5, *p* = 0.02) and 2,000 m time trial (*t* = 3.4, *p* < 0.01), indicating greater slopes for the LIHV training condition. No time by training conditions were found for other outcomes. Table 2 summarizes all model parameters and test statistics. Figures 2, 3 illustrates the distribution of individual data and the model (i.e., predicted) group data based on parametric bootstrapping.

**TABLE 2:** Linear mixed-effects models for between-subject analyses.

Parameter	Estimate	SE	*t*	*p*
**Bench pull average power (W⋅kg^–1^)**				
N (MILV/LIHV)	15/10			
Intercept	1.070	0.051	20.8	<0.001*
Condition	0.048	0.081	0.59	0.563
Time (month)	0.022	0.005	4.21	<0.001*
Time × condition (month)	−0.021	0.008	−2.45	0.023*
**Shot throw (m)**				
N (HI/LI)	16/10			
Intercept	8.07	0.376	21.5	<0.001*
Condition	1.38	0.606	2.27	0.032*
Time (month)	0.113	0.025	4.50	<0.001*
Time × condition (month)	−0.0815	0.040	−2.01	0.055
**Hand grip strength (N)**				
N (MILV/LIHV)	23/14			
Intercept	262	10.5	23.3	<0.001*
Condition	23.7	16.8	0.91	0.369
Time (month)	2.69	0.57	4.72	<0.001*
Time × condition (month)	0.54	0.94	0.57	0.567
**Skeletal muscle mass (kg)**				
N (MILV/LIHV)	24/14			
Intercept	24.8	1.08	23.0	<0.001*
Condition	0.426	1.78	0.24	0.812
Time (month)	0.283	0.027	10.3	<0.001*
Time × condition (month)	−0.006	0.045	−0.13	0.895
**Time trial 250 m (s)**				
N (MILV/LIHV)	10/9			
Intercept	68.1	1.51	45.0	<0.001*
Condition	5.58	2.53	2.20	0.042*
Time (month)	0.05	0.06	0.86	0.404
Discipline	1.47	2.52	0.58	0.568
Time × condition (month)	0.05	0.08	0.56	0.581
**Time trial 2,000 m (s)**				
N (MILV/LIHV)	9/10			
Intercept	652	13.7	47.6	<0.001*
Condition	46.8	21.1	2.21	0.038*
Time (month)	−2.28	1.96	−1.16	0.260
Discipline	52.8	19.5	2.70	0.016*
Time × condition (month)	9.16	2.70	3.40	0.003*

The time × condition parameter shows the difference between training conditions over time (i.e., per month). MILV, moderate intensity, low volume strength training condition; LIHV, low intensity, high volume strength training condition. The reference condition is LIHV. The reference discipline is canoe. Asterisks (*) flag statistical significant (p < 0.05) effects.

### Within-Subject Analysis

**FIGURE 4:**
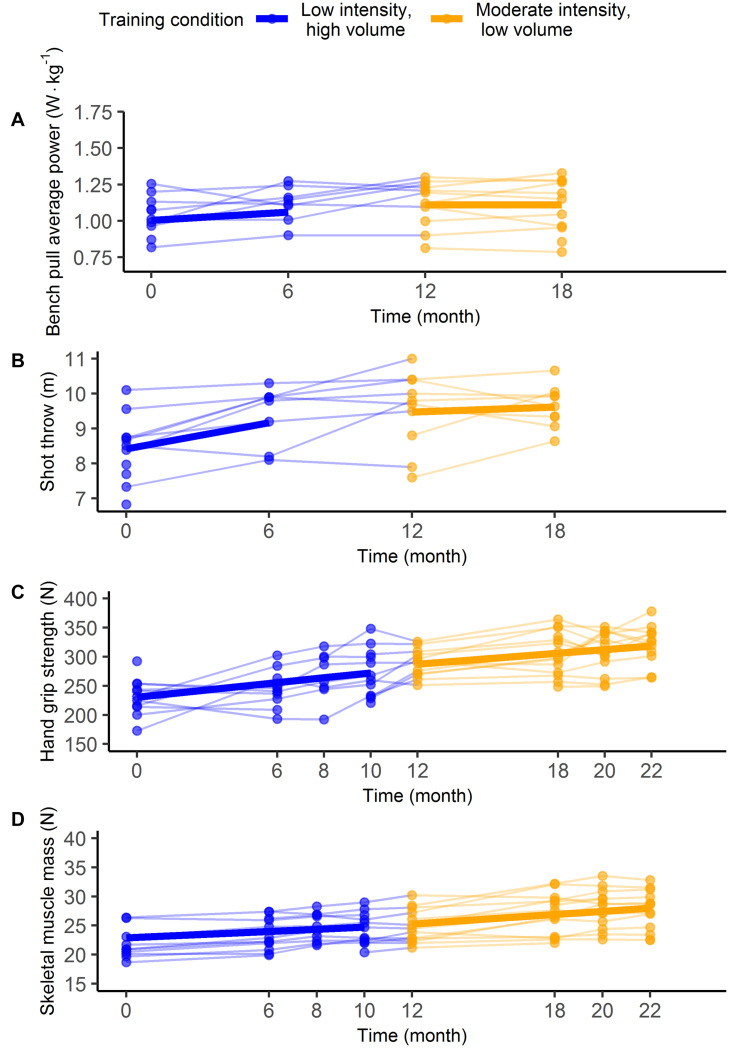
Individual data (thin lines) and model estimates (thick lines) of the within-subject analyses for the outcomes bench pull average power **(A)**, shot throw **(B)**, hand grip strength **(C)**, and skeletal muscle mass **(D)**.

Outcomes that significantly increased over time included the 2,000 m time trial, shot throw, handgrip strength, and skeletal muscle mass (*t* ≥ 2.1, *p* < 0.05), but no main effects of time were found for the outcomes bench pull average power and the 250 m time trial. A significant time by training condition interaction was present for the 2,000 m time trial (*t* = 2.8, *p* = 0.01), indicating a greater slope for the LIHV training condition. No time by training conditions were found for other outcomes. Table 3 summarizes all model parameters and test statistics. Figures 3, 4 illustrates the distribution of individual data and the model (i.e., predicted) group data based on parametric bootstrapping.

**TABLE 3:** Linear mixed-effects models for within-subject analyses.

Parameter	Estimate	SE	*t*	*p*
**Bench pull average power (W⋅kg^–1^)**				
N	12			
Intercept	1.01	0.050	20.1	<0.001*
Condition	0.105	0.038	2.76	0.011*
Time (month)	0.010	0.007	1.44	0.162
Time × condition (month)	−0.010	0.009	−1.05	0.306
**Shot throw (m)**				
N	11			
Intercept	8.41	0.279	30.2	<0.001*
Condition	1.06	0.219	4.82	<0.001*
Time (month)	0.127	0.0395	3.22	0.004*
Time × condition (month)	−0.103	0.0565	−1.83	0.080
**Hand grip strength (N)**				
N	14			
Intercept	231	9.49	24.3	<0.001*
Condition	56.5	8.71	6.49	<0.001*
Time (month)	4.15	0.88	4.71	<0.001*
Time × condition (month)	−1.01	1.22	−0.83	0.408
**Skeletal muscle mass (kg)**				
N	14			
Intercept	22.9	0.848	27.0	<0.001*
Condition	2.34	0.484	4.83	<0.001*
Time (month)	0.185	0.05	3.71	<0.001*
Time × condition (month)	0.0914	0.067	1.36	0.176
**Time trial 250 m (s)**				
N	11			
Intercept	74.3	2.27	32.8	<0.001*
Condition	−3.15	1.39	−2.27	0.032*
Time (month)	0.428	0.257	1.67	0.108
Discipline	5.28	2.5	2.11	0.062
Time × condition	−0.369	0.345	−1.07	0.296
**Time trial 2,000 m (s)**				
N	12			
Intercept	734	24.2	30.3	<0.001*
Condition	−61.8	19.8	−3.12	0.004*
Time (month)	−7.78	3.78	−2.06	0.049*
Discipline	90.8	24.8	3.66	0.005*
Time × condition (month)	13.90	5.02	2.77	0.010*

The time × condition parameter shows the difference between training conditions over time (i.e., per month). The reference condition is low intensity, high volume strength training condition (LIHV). The reference discipline is canoe. Asterisks (*) flag statistical significant (p < 0.05) effects.

### Race Time Prediction

Inserting the LME coefficients for bench pull power and skeletal muscle mass into previous regression models (Gäbler et al., 2021) predict that 500 m race time improved monthly by 0.37–0.45 s during the MILV condition and by 0.67–1.35 s during the LIHV condition. The models predict that 2,000 m race time increased monthly by 1.90–2.15 s during the MILV condition and by 2.73–5.30 s during the LIHV condition. The ranges indicate the different predicted values whether a within-subject or between-subject design is used to obtain the LME coefficients.

## Discussion

The aim of this study was to compare the effects of MILV vs. LIHV strength training on sport-specific performance, measures of muscular fitness, and skeletal muscle mass in young kayakers and canoeists. In the present study, we evaluated the hypothesis that training adaptations are specific to the intensity and volume of the training. The results suggest greater improvements during LIHV vs. MILV strength training in 2,000 m performance and average bench pull power, a proxy of on-water performance.

### Sport-Specific Performance

Our results suggest that the intensity and volume of strength training affect canoe and kayak performance over 2,000 m, but no effect was observed over 250 m. LIHV compared with MILV strength training produced favorable results in time trials of 2,000 m and both 500 and 2,000 m race time predicted from physical fitness and anthropometric variables. The different methods suggested that LIHV compared with MILV strength training improves 2,000 m time by 0.8–13.9 s per month. Unfortunately, to the best of our knowledge, there have been no training studies that directly assessed kayak or canoe race time. It appears to be challenging to quantify performance in sports and it is described as a multidimensional construct (Elferink-Gemser et al., 2007). As a result, many physical exercise intervention studies evaluate correlates of performance, but not the actual performance itself. Our approach allowed the estimation of training effects on kayak/canoe performance through combining differing methods. Although it was not possible to control external factors that act on the boat, we observed excellent test-retest reliability (ICC > 0.93) for the time trials. Besides, the predicted values agreed at least directionally with the performance observed during the time trials. The directions of the effects also agreed for the within- and between-subject analyses, strengthening the conclusion that the LIHV compared with the MILV strength training improved 2,000 m kayaking/canoeing performance.

### Muscular Fitness

The most pronounced effect of strength training condition was observed in muscular endurance, measured with a 2-min bench pull endurance test and normalized to body mass. Participants had a greater increase in muscular endurance during the LIHV vs. MILV condition. We did not find an effect of training condition on muscle strength, quantified as the maximum force during isometric contraction of the hand, nor on muscle power, quantified as the underhand shot throw. However, there was a trend (*p* = 0.08) towards greater muscle power improvements in participants during the LIHV vs. MILV training condition. In previous work, 8-year-old children improved muscle strength and muscular endurance more when they performed exercises with a greater number of repetitions and a lower intensity compared with a smaller number of repetitions and a higher intensity (Faigenbaum et al., 1999). Also, untrained female adults did not demonstrate intensity/volume-specific adaptations along the strength/endurance continuum (i.e., intensity/number of repetitions) (Stone and Coulter, 1994). These observations suggest that the principle of training specificity might not always apply to the intensity and volume of strength training. On the other hand, perhaps the training intensity was not specific to the intensity in the investigated outcomes.

According to the principle of training specificity (Häkkinen et al., 1989), MILV strength training is a more specific training stimulus to improve muscle strength, and perhaps power, than LIHV strength training. The greatest increases in muscle strength were observed in studies using intensities above 85% of 1-RM (Peterson et al., 2004; Lesinski et al., 2016). The greatest increases in lower body muscle power were observed in studies using plyometric training (Harries et al., 2012). There is no straightforward method to translate plyometric exercise to a percentage of 1-RM. However, it is known that the greatest power output can be achieved at intensities somewhere between 40 and 70% of 1-RM (Siegel et al., 2002) and that strength increases are velocity specific (Behm and Sale, 1993). If we critically evaluate the exercise protocol, it seems that the intensity of training (70–80% 1-RM) may have been too low to optimize muscle strength adaptations, and too high to optimize muscle power adaptations. The LIHV condition allowed a greater muscle contraction velocity than the MILV condition, which could explain the slightly greater power adaptations that were observed in athletes performing the LIHV condition. As maximum muscle strength is a predictor of canoe and kayak performance, it may be beneficial for canoe and kayak athletes to perform exercises at intensities higher than in this study (<85% 1-RM). However, we note that pediatric strength and conditioning specialists frequently argue that children and adolescents should not perform strength training at intensities above 80% of 1-RM if their strength training skill competency is not adequately developed (Lesinski et al., 2016; Gäbler and Granacher, 2019).

### Skeletal Muscle Mass

Monthly increases in skeletal muscle mass ranged between 0.19 and 0.28 kg, irrespective of the training condition (Tables 2, 3). There is no consensus (Behm et al., 2008) and only indirect evidence (Legerlotz et al., 2016) that strength training produces muscle hypertrophy in adolescents. However, it was suggested that the changes in hormonal status that take place around the timing of peak height velocity facilitate training-induced muscle hypertrophy in adolescents (Lloyd and Oliver, 2012). Still, it remains difficult to separate maturational processes from training adaptations. Reference values suggest that the monthly increases in muscle mass in 50th percentile of 13-year boys and girls are, respectively, 0.179 and 0.087 kg (McCarthy et al., 2002). The increase in skeletal muscle mass observed in our training was above these reference values, implying that the training induced muscle hypertrophy. A lack of a time by condition interaction suggests that muscle hypertrophy was independent of training condition. The proportion of boys, who, compared with girls, increased their skeletal muscle mass at a greater rate (McCarthy et al., 2014), was higher in the LIHV condition of the between-subject analysis. Furthermore, the within-subject analysis revealed that athletes completing both training regimes vs. a single training condition experienced a 0.09 kg greater rate of increase in muscle mass. Therefore, the possibility should not be ruled out that MILV may result in larger muscle hypertrophy increases compared with LIHV. Although this was not statistically significant, it may be a meaningful difference considering that each additional kilogram of skeletal muscle mass predicts a 1.0–1.3 s faster 500 m race time and a 6.6–9.6 s decrease in 2,000 m race time (Gäbler et al., 2021).

During the high intensity training condition, athletes performed the prone bench pull and bench press exercises at intensities between 70 and 80% of 1-RM. The ACSM position stand recommends training at an intensity above 60% of 1-RM to promote muscle hypertrophy (Ratamess et al., 2009). This theoretically follows from the size principle in motor unit recruitment (Henneman et al., 1965) and indeed, there seems to be a dose-response relationship between intensity and hypertrophic response, at least in untrained adult males (Campos et al., 2002). After 8 weeks of training, only the groups that performed a low (Forbes et al., 2009; Hamano et al., 2015; Gäbler et al., 2021) or intermediate (Peterson et al., 2004; Lesinski et al., 2016; Nilsson and Rosdahl, 2016), but not high (Stone and Coulter, 1994; Faigenbaum et al., 1999; Elferink-Gemser et al., 2007; Cousineau and Chartier, 2010; Harries et al., 2012; Barr et al., 2013; Bates et al., 2015; Kuznetsova et al., 2017; R Core Team, 2020) number of repetitions increased their muscle fiber cross sectional area. The intensity was progressed in such a way that exercise failure occurred within the prescribed number of repetitions. Translated to a percentage of 1-RM (Shimano et al., 2006), the low, intermediate, and high repetition groups trained at an intensity of approximately >90, 80, and <60% 1-RM, respectively. More recent evidence suggested, however, that muscle hypertrophy can be achieved in untrained subjects with an exercise intensity as low as 20% of 1-RM when performing the exercise until failure (Lasevicius et al., 2018). At intensities of 40% and higher, relative improvement in muscle CSA was almost numerically equal, ranging between 19.5 and 20.5%. Although we cannot draw any firm conclusions about the within subject analysis, the main effect of time and interaction term suggest that MILV strength training might increase muscle hypertrophy in young kayakers and canoeists.

Endurance training potentially interferes with the development of muscle mass, strength and power (Wilson et al., 2012). Interference is hypothesized to occur when strength and endurance training both target specific adaptations at the muscular level (i.e., mitochondrial growth and muscle hypertrophy) (Docherty and Sporer, 2000). In the present study, endurance training consisted mainly of low intensity running and swimming and mainly targeted central (i.e., cardiovascular) adaptations. It seems, therefore, unlikely that endurance training interfered with adaptations to strength training.

### Strengths and Limitations

Studies in highly trained young individuals are scarce, particularly over a prolonged period of time. To the best of our knowledge, this is the first controlled study that evaluated training effects on (proxies of) kayak or canoe performance. The duration and population of this study make it unique, but also provide some difficulties and limitations that should be addressed. The athletes trained in predetermined groups across 2 years. Therefore, it was not feasible to randomize participants to training conditions which increases the risk of bias. Moreover, differences between seasons when measurements were performed (i.e., weather) may have influenced the results. With a maturing study population, it remains difficult to separate training adaptations from growth and maturation.

Despite these limitations, it should be pointed out here that the young, highly trained subjects in the present study were recruited from a small, but homogeneous overall population (i.e., youth athletes) in terms of fitness and expertise level. Furthermore, we tried to manage as adequately as possible the incomplete data by using LME models. To mediate potential bias, we incorporated both the between- and within-subject analysis, thus combining the advantages of both designs (Charness et al., 2012). Other researchers, in particular those working with highly trained populations, should consider these methods when facing similar challenges.

## Conclusion

In conclusion, young sprint kayakers and canoeists up to 14 years are advised to perform strength training at low intensity and high volume to improve potentially on-water performance. LIHV vs. MILV strength training improved long distance (i.e., 2,000 m time trial) kayak/canoe sprint performance and muscular endurance (i.e., average bench pull power), but not muscle strength, power, or skeletal muscle mass. Our data predict that the strength training condition translates to a difference (LIHV minus MILV) in monthly race time changes ranging from −0.05 to +0.37 s for 250 m, −0.3 to −0.9 s for 500 m, and −0.8 to −13.9 for 2,000 m.

## Data Availability Statement

The raw data supporting the conclusions of this article will be made available by the authors, without undue reservation.

## Ethics Statement

The studies involving human participants were reviewed and approved by the University of Potsdam (University of Potsdam: submission No. 5/2014). Written informed consent to participate in this study was provided by the participants’ legal guardian/next of kin.

## Author Contributions

MG, OP, TW, TH, and UG contributed to conception and design of the study. MG organized the database. MG and HB performed the statistical analysis. MG, OP, TW, TH, ME-G, and UG contributed substantially to the interpretation of the data. MG wrote the first draft of the manuscript. HB wrote the statistical analysis section of the manuscript. All authors contributed to manuscript revision, read, and approved the submitted version.

## Conflict of Interest

The authors declare that the research was conducted in the absence of any commercial or financial relationships that could be construed as a potential conflict of interest.
